# Jump step - a community based participatory approach to physical activity & mental wellness

**DOI:** 10.1186/s12888-017-1476-y

**Published:** 2017-08-31

**Authors:** Joanie Sims-Gould, Sara Vazirian, Neville Li, Ronald Remick, Karim Khan

**Affiliations:** 1 Department of Family Practice, University of British Columbia, 793-2635 Laurel Street, Vancouver, BC V5Z 1M9 Canada; 2 University of British Columbia, Center for Hip Health and Mobility, 2635 Laurel Street, Vancouver, BC V5Z 1M9 Canada; 3 University of British Columbia, Center for Hip Health and Mobility, 2635 Laurel Street, Vancouver, BC V5Z 1M9 Canada; 4 Mood Disorders Association of BC, 1450 - 605 Robson Street, Vancouver, BC V6B 5J3 Canada; 5 Department of Family Practice, University of British Columbia, 5950 University Blvd, Vancouver, BC V6T 1Z3 Canada

**Keywords:** Physical activity, Depression, Anxiety, Mental health, Group medical visits, Exercise

## Abstract

**Background:**

There is a physical inactivity pandemic around the world despite the known benefits of engaging in physical activity. This is true for individuals who would receive notable benefits from physical activity, in particular those with mood disorders. In this study, we explored the factors that facilitate and impede engagement in physical activity for individuals with a mood disorder. The intent was to understand the key features of a community based physical activity program for these individuals.

**Methods:**

We recruited and interviewed 24 participants older than 18 with Major Depressive Disorder or Bipolar II. The interviews were conducted by peer researchers. The interviews were transcribed and analyzed using NVivo 10™. Thematic analysis was used to analyze the data.

**Results:**

The facilitators to physical activity include being socially connected with family and friends, building a routine in daily life, and exposure to nature. The barriers to physical activity include the inability to build a routine owing to a mood disorder, and high cost. The ideal exercise program comprises a variety of light-to-moderate activities, offers the opportunity to connect with other participants with a mood disorder, and brings participants to nature. The average age of our participants was 52 which could have influenced the preferred level of intensity.

**Conclusion:**

The individuals in this study felt that the key features of a physical activity program for individuals with a mood disorder must utilize a social network approach, take into account the preferences of potential participants, and incorporate nature (both green and blue spaces) as a health promotion resource.

## Background

Globally, physical *inactivity* is “pandemic” [1]. Exercise or regular physical activity is often described as a “polypill” [2] – a single treatment with at least 13 documented health benefits. Only 150 min a week of moderate exercise is required to receive the health benefits of physical activity, yet globally around 31% of adults aged 15 and over were reported to be insufficiently active in 2008 (men 28% and women 34%) with approximately 3.2 million deaths each year linked with insufficient physical activity [3]. Physical activity is relatively free of adverse side effects and can be a low-cost treatment, especially when compared to pharmaceutical agents. It is known that the promotion of physical activity is complex with a myriad of influential factors at play, including patient activation, the built environment, and one’s socioeconomic and demographic status to name just a few [4–9].

Mood disorders (e.g., depression, anxiety) are a worldwide health epidemic resulting in significant morbidity and mortality [10, 11]. The rate of physical inactivity is even higher in these individuals as compared to the general population putting them at a higher risk of cardiovascular events [12]. Nearly two thirds of people suffering from Major Depressive Disorder (MDD) do not meet the physical activity recommendations [13]. The evidence is unequivocal that in terms of treating a mood disorder, regular physical activity can be as effective as either of the more widely known and used treatments – antidepressant medication and/or cognitive psychotherapy [14, 15]. In fact, a large Cochrane review of exercise as a treatment for depression [14] recommended that future research move beyond efficacy studies and begin to examine what types of exercise and the number and duration of sessions are of most benefit to patients with depression. There is recent research showing that individuals with depression prefer to be in a walking program multiple times a week [16], and researchers trying to find effective ways to promote physical activity as an intervention for individuals with psychosis [17]. A qualitative study with individuals with depression finds that they view physical activity as an effective treatment, but the desired format of the exercise and the barriers to participation are varied [18]. To that end we completed a pilot study of a group based intervention for patients with depression (14 weekly 2 h sessions, combining 1 h of physical activity led by a personal trainer with an hour of group medical visit led by a psychiatrist) demonstrating improvement in patients’ physical activity levels as well as decreases in their levels of depression and anxiety [19].

In building this pilot study (focused on the effectiveness of physical activity for persons with depression and anxiety), the next step was to examine individual experiences to gain a comprehensive understanding of the barriers and facilitators to physical activity for individuals with a mood disorder. This is the focus of the present study.

## Methods

Community-based research in public health focuses on social, structural, and physical environmental inequities through active involvement of community members, organizational representatives, and researchers in all aspects of the research process. Partners contribute their expertise to enhance understanding of a given phenomenon and to integrate the knowledge gained with action to benefit the community involved [20]. A community based participatory research approach was chosen for this project as it is a method that increases the relevance of the data collected by engaging the community members with the lived experience of the problem under research; it promotes the sustainability of the program designed as it has taken into account the actual needs of the service recipients; and the community members also play a significant role in the design and execution of the project [20–22]. Taking these benefits into account, we recruited participants with a diagnosis of depression and/or bipolar disorder in this study. These individuals served as advisors in the design and data collection of the study and as participants as per the participatory approach.

### Participant overview

A sample of 24 participants (average age 52 years) with a DSM-5 diagnosis of Major Depressive Disorder (MDD) (*N* = 18) or Bipolar II depression (BPII) (*N* = 6) [19] were recruited from the Lower Mainland, British Columbia. The inclusion criteria were: 1) age over 18 years; 2) confirmed psychiatric diagnosis of MDD and/or BPII; 3) community-dwelling and able to attend Group Medical Visits (GMVs); 4) able to comply with scheduled visits, treatment plan, and other procedures; 5) fluent in English; 6) able to provide signed and dated written informed consent; and 7) able to walk independently. The exclusion criteria included: 1) active psychotic symptoms; and 2) a primary, active diagnosis of substance abuse.

### Recruitment

The University of British Columbia (UBC) Behavioral Ethics Board provided ethical approval prior to the study’s commencement. We recruited the participants via four methods. First, we displayed posters in the waiting area of the Mood Disorders Association of BC (MDABC), the psychiatric service areas of major hospitals in the Lower Mainland, areas visible to potential volunteers within related service organizations such as the Canadian Mental Health Association (CMHA), coffee shops, community centres, and YMCAs. Second, we posted an advertisement on the websites and distributed a message via the list-serves of related service organizations such as the MDABC, the BC Psychiatric Association, BC Mental Health & Addiction Services, Health Authority Mental Health Services, and the CMHA. Third, we recruited through word-of-mouth at the MDABC and other service agencies. MDABC psychiatrists and staff verbally informed clients of the opportunity to participate with the assurance that participation or non-participation would not affect their access to or the quality of psychiatric care and other services offered by the MDABC. Fourth, we posted messages on social media such as Facebook and Twitter.

### The use of peer researchers

The use of peer researchers was an integral component of our community based participatory research design. Peer researchers have been used in a variety of community based participatory studies to decrease the power differential often created by the traditional researcher-participant approach [23, 24]. Peer researchers were recruited and trained to conduct the participant interviews. In the initial meetings of our community advisory committee, comprised of individuals with a mood disorder and researchers, it was decided that peer researchers would be able to relate to participants and more likely to elicit authentic responses. Peer researchers met the same inclusion/exclusion criteria as study participants and were recruited in the same format. In two half-day workshops, UBC investigators trained the peer researchers on the purpose of the study and in interviewing techniques. The peer researchers had the opportunity to practice their interview skills and receive feedback after each interview conducted. They were paid a small honorarium for their time and reimbursed for public transport/parking expenses. They received gift cards and were entered in a draw to win one of four $150 gift cards. The training workshops took place in September 2014 and the interviews occurred between November 2014 and October 2015.

### Data collection

Interviews were conducted one-on-one in a private interview room at a UBC senate approved research facility (Center for Hip Health and Mobility (CHHM)) and lasted between 90 and 120 min. Three interviewers were present at each interview - two peer researchers and one research assistant (who assisted with note taking). The interview guide was designed by UBC investigators and peer researchers and focused on participant’s history with physical activity and perceived facilitators and barriers to being physically active. Table 1 gives an example of the types of questions in the interview guide.

**Table 1:** Sample interview questions

A - Examples of questions regarding physical activity
1. Describe what a typical day looks like for you? Is physical activity a part of your typical day?
2. How much time per week are you in nature/the outdoors? Why or why not?
B - Examples of questions regarding participants’ coping strategies
1. What are you currently doing to deal with your anxiety/mood disorder? Is physical activity a part of it?
2. How effective do you find your current treatment options in improving your quality of life? Why or why not?
C - Examples of questions regarding barriers/facilitators to becoming more physically active
1. Is physical activity part of your ideal day? Why or why not? What are some of the barriers or facilitators to be physically active?
D - Examples of questions regarding ideal physical activity program
1. If you were to design the PERFECT physical activity program for YOU, what would it look like and feel like?
2. What would some key features be in terms of time, location, duration, types of physical activity, etc.?

### Participant demographics

Table 2 highlights key demographic characteristics of our participants.

**Table 2:** Participant demographics

Participant	Age	Gender (Male/Female)	Daily Average Steps
001	43	M	4176
002	44	F	4461
003	52	F	4946
004	45	F	8597
005	58	F	11,343
006	59	F	7255
007	56	M	2165
008	46	M	7136
009	61	M	1562
010	54	F	3125
011	53	F	2952
012	66	F	2297
013	55	F	752
014	50	M	3131
015	47	M	8396
016	52	F	4130
017	54	F	8118
018	66	M	6126
019	75	F	6262
020	57	M	3450
021	53	F	4563
022	20	F	3592
023	45	F	8562
024	50	F	5045
	Average = 52.5	M = 8 F = 16	Average = 5089

### Data analysis

Interviews were audio recorded and transcribed verbatim using a professional transcription service. We then removed participant identifiers and replaced real names with pseudonyms. Two members read the transcripts to come up with a coding framework. They then had a discussion of the frameworks and drafted a preliminary framework. It was passed along to four other investigators for feedback and suggestions. A final coding framework was developed and then one team member coded all 24 transcripts using NVivo 10™ software. We used thematic analysis [25] and in order to enhance rigor, the team met repeatedly to discuss emerging themes and to provide feedback. Peer researchers were also consulted in the process. We also conducted member checking by presenting the results to a group of peer researchers and participants and solicited feedback to “stay true to the data.” We adhered to COREQ guidelines in this study.

## Results

Three main questions we were trying to answer were: facilitators and barriers to physical and mental health; personal strategies to improve health and quality of life and the features of an ideal physical activity program for individuals with mood disorders. The themes that emerged as a response to our main questions are discussed below in more details.

### Facilitators and barriers to physical and mental health

Participants discussed at length facilitators and barriers to their physical and mental health. Facilitators to positive physical and mental health included the availability and presence of social support and the opportunity to engage in social activities. All participants identified their mood disorder diagnosis as being a threat to both their physical and mental health. Participants also spoke about barriers to engaging in physical activity despite understanding the benefits of being active.

#### Facilitators

The most common themes are 1) support from spouse, family or friends, and 2) social activities.

##### Support from spouse, family or friends

The vast majority of participants said that their family and friends gave them the support, encouragement, and practical help around daily tasks so that they could improve their own health. For example,

*My husband is very supportive. For a long time he was making almost all the meals and stuff like that. But he has a really busy job, and the advantage of [for] me? I can be at my best, or close to. If I could be fully functioning and doing a bunch of stuff that makes his life better too. (P003, Female, 52)*

##### Social activities

Among the participants, getting involved in social activities with other people, as opposed to spending the day by oneself, was a desired way to improve their own health.

*Q: Do you see that as a part of your treatment, the exercise?*

*A: Oh, yeah, definitely. Definitely helps. Socializing with people. It helps.*

*Q: And you see socialization as part of your treatment, right?*

*A: Yeah, I think socializing is just a part of life. (P009, Male, 61)*

#### Barriers

The emergent theme in this regard is the mood disorder as a formidable barrier in itself creating a vicious cycle:

*Getting out of bed is hard. I wake up and I feel almost drugged, and it’s sort of…pulling myself out of the swamp. It’s a comfortable swamp of semi-consciousness. So if I can make myself get up, I’m fine. (P006, Female, 59)*

In addition, participants spoke about barriers to engaging in physical activity despite the known benefits. The most common barrier to engaging in activity was cost. Participants spoke about disruptions to income and lack of financial means to engaging in activity. One participant stated:

*A: But-- ‘cause I was really enjoying going to yoga, but I’ve taken, like, I’ve paused my membership because I have no income right now.*

*Q: So finances are definitely--*

*A: Finances are a huge stressor at the moment. (P024, Female, 50)*

### Personal strategies to improve health and quality of life

Despite challenges to physical and mental health and engagement in physical activity, participants devised a number of ways to improve their own health. These strategies were highly personal and diverse. However, there were certain common themes which included getting out of the house; building a routine; and, connecting with nature.

#### Getting out of the home/house

A consensus among participants was that not being confined to the home was very important. They often mentioned being “overly-stimulated” and “overwhelmed” by the outside world because of the number of people, noise and even daylight. Consequently, many participants stayed home most of the time. Participants recognized this as a vicious cycle that worsens their mental health, turning something as simple as going outside of the house a coping strategy.

*Q: What do you believe are the most important things you could be doing to improve your mental health? The most important things. So not everything. Just the most important you could do.*

*A: Just trying to get out. Trying to get out every day probably, trying to put some more structure in without overdoing it, like, without committing to too much. (P003, Female, 52)*

#### Building a routine/structure in daily life

Some participants mentioned that having a routine with expected activities helped them manage their health. Typically, many of these routines involve getting out of the house for seeing friends and buying groceries:

*Getting up every day and kind of having a little bit of a plan for what I need to accomplish. And also just, like, having a certain built-in structure of my son goes to school. I can’t let my own depression, anxiety, mental health issues affect his life. … I need to make sure that his day is structured, but because I need to do this, it gives me a structure for my day. (P005, Female, 58)*

#### Connecting with nature

When probed about their relationship with nature, a number of participants said that nature calmed their nerves:

*I love nature. We’ve got-- as I say, we have a mountain cabin. I love being up there. … Breathing room, fresh air, peace and quiet. And then the types of activities outside, things like for me, chopping wood, building projects, handyman type work. Taking care of the boat, those sorts of things I enjoy. (P007, Male, 56)*

Closely related to the enjoyment and serenity that nature brings, some participants described nature as “natural” for human beings. In other words, humans ought to be outdoors in nature more to achieve a natural state of mind:

*A: Yeah. And nature’s great ‘cause you get differentIt’s [nature’s] good for you. I actually happen to like broccoli and I enjoy it. But I actually really think that people need to get out of an urban environment and into nature-- ‘cause sometimes I think it’s really unnatural for everybody to live so shoulder to shoulder.*

*Q: So nature also includes space for everybody.*

*A: Yeah. And nature’s great ‘cause you get different smells. You get, like, rich organic smells and you get crisp smells and you’re not smelling toxins and diesel. (P005, Female, 58)*

Participants also noted that connecting with nature increased the likelihood of engaging in physical activity:

*But also because quite often when I’m in the outdoors I’m being physically active. Like, I’m not just-- I’m not being rolled to a park and sat there or something. So it’s usually connected with activity. And just-- I find it’s very serene to the point where something like a cell-phone call or something just seems to disrupt the serenity of-- yeah, of the moment. (P008, Male, 46)*

### Features of an ideal physical activity program

Based on participant’s discussions of physical activity, facilitators and barriers to physical activity we were able to distill a list of key features of an ideal physical activity program for individuals with mood disorders. These key features included: the importance of peer support (a community of individuals with similar diagnoses); varied activities; and recommendations on intensity, duration and time of day, and cost.

#### Peer support

Participants spoke at length about the importance of being part of a caring community with encouragement as a central tenet. One participant stated:

*If there was…if I was part of a group, to see that I’m-- or giving me encouragement, whether it be people that you’re working out with, or an instructor, coach type thing, positive … positive reinforcement. (P002, Female, 44)*

In addition to a caring community, participants also identified that a program specifically for individuals with a mood disorder would have the potential to create a sense of community and sharing with other people who were “in the same boat”. One participant summarized this by stating:

*To share the place with other people with the same illness like me. Because they don’t go ah, they don’t [sic] going to judge me. And I am not going to judge them. So we are in the same boat, and we are trying to get the same reach. (P013, Female, 55)*

#### Varied activities

To pique participants’ interest and to offer the greatest health benefits, a number of participants suggested an all-around fitness program that would include a cardio component, weight training, and activities deemed enjoyable, such as yoga. Another key factor of a perfect program is to take the attendees’ interests into account. Many participants mentioned walking, yoga, swimming, and weight lifting as the most desired exercise options because they were fun, economically feasible, and activities they had done previously.

*Yeah, you can’t pigeonhole people into something. Doesn’t work. If you try to get somebody who doesn’t like working out, working out to try and find out if it’s going to improve their mental health, they’re going to tell you … why are you making me do this? I don’t want to do in the first place. (P015, Male, 47)*

#### Intensity, duration, time, cost

Participants also offered advice on the structure of a perfect physical activity program, despite personal preferences; there are general commonalities across all the responses. The preferred frequency of an ideal program is 2–3 times per week; the intensity is typically light to moderate; with a duration of 30–60 min. We understand that the average age of our participants might have influenced their preference in terms of their preferred intensity. Most participants prefer a session between morning and early evening, with no participant saying that they want a program offered at night. As identified, lack of financial resources is a barrier to participation in physical activity. Therefore, it is not surprising to hear from participants that the availability of a free or low-cost program would go a long way to ensuring program success. The ideal scenario is that a program would be offered free-of-charge.

*I mean, if it was set up on a six-month program at a fairly reasonable cost to get me started, something like that. Money is always a barrier, but in my case, it’s a-- it might be a barrier, but it’s not an excuse. (P020, Male, 57)*

## Discussion

The goals of this study were to understand the engagement in physical activity among individuals with a mood disorder, the facilitators and/or barriers for them to become physically active and the key features of a physical activity program specific to their needs.

The majority of the participants were not content with their current level of physical activity. There was a general understanding of the importance of physical activity and its positive impact on their mental as well as overall health. Some participants did consider it one of the coping strategies and even regarded it as a part of their treatment.

The facilitators of and barriers to physical and mental health closely resembled participants’ discussions of engagement in physical activity. For example, participants spoke at length about the importance of social support; they also discussed supportive features as being key to the success of a physical activity program. This emphasis on social support is in alignment with qualitative research on people with first-episode psychosis, in which persons with psychosis are more engaged in moderate-to-vigorous exercise with a social network [26]. Participants were very clear about strategies they employed to promote and improve their own health and to enhance their quality of life. These strategies included building a daily routine to “get out of the house” such as walking their children to school. These results echo the findings from a recent study on individuals with longer-term depression. In the study, Chambers et al. [27] found that an individualized model with more choices and control facilitates self-management of depression. Our participants also benefited from their own choice of physical activity and daily exercises.

Levula et al. [28] stated in their study that social network factors are associated with mental health across the three life stages of adolescence, adulthood and old age, and that their findings have implications for mental health intervention design. Many of our participants’ coping strategies were related to building a social network to avoid social isolation and other negative effects of mental illnesses. Their established strategies provided key insights to the design of an ideal physical activity program for individuals with a mood disorder, and the incorporation of different ways for participants to develop social connections should not be neglected. The program can borrow the concept of a social network approach in community mental health nursing that has been in practice since the 1970s [29]. In this approach, the clinician utilizes the social network of the person with mental health issues to solve problems collectively. Applying this strategy to exercise interventions for patients with mood disorders, we can gather the friends, family and acquaintances of an individual with a mood disorder to plan and solve problems together, to strengthen a sense of solidarity and to lessen the feeling of aloneness. Specifically, the participants in the program can act as peer support during both good and challenging times, the family or friends can offer encouragement and a reminder for the individual with a mood disorder to engage in consistent physical activity, the sports coaches can contribute their expertise on exercise techniques and be a source of accountability, and the clinicians such as psychiatrists or psychologists can educate the participants on the scientific ways to manage their symptoms. Previous research findings have pointed to the support of mental health staff and the structure of the exercise program to be enabling factors to participation among individuals with a mood disorder [30].

Researchers have explored the relationship between natural environments or green environments and mental health in fields ranging from epidemiology [31], psychology [32] and geography [33]. All of these studies have demonstrated that the natural environments, whether proximity to it and/or engaging in physical activity within it, are effective in boosting a person’s mental health. Finlay et al. [34] found out that both green (e.g., parks, gardens) and blue (e.g., lakes, oceans) spaces have therapeutic effects on older adults. These “therapeutic landscapes” enhance their physical, mental and spiritual health and wellbeing as well as elicit feelings of renewal, restoration and spiritual connectedness. The authors suggested that public health strategists and urban planners should devise ways to utilize nature as a health promotion resource. In a 2016 study [35], outdoor exercises were proved to provide more affective improvements and enhance psychological well-being than indoor exercises to people with depression. In our study, participants’ accounts confirmed the cathartic effects of nature or the outdoors and an increased level of physical activity coming from being in nature. From this, we suggest that incorporating nature or the outdoors in a physical activity program for individuals with a mood disorder is essential.

Within the literature there is relatively recent evidence that physical activity is an effective treatment for mood disorders as opposed to only as a treatment to improve physical health [36–38]. Vancampfort et al. [39] developed a list of ten questions for researchers to take into consideration when attempting to promote physical activity (PA) for people with bipolar disorders (BD). Examples of these questions include i) what are the key barriers to PA among people with BD? ii) what are the most effective motivational strategies for ensuring PA adoption and maintenance in BD? and iii) If one treatment goal is increased physical activity, what type of professionals are needed as part of a multidisciplinary team? Besides the actual physical activity program, other factors may also be pivotal to the successful promotion of PA. For example, one quantitative study conducted in Australia found that among middle-aged and older adults, in addition to the physical activity programs, personal safety, the neighborhood environment, and social support from family and friends also improved mental health-related quality of life [40]. In our study, participants confirmed and extended these previous findings and offered detailed information on how to create a physical activity program that is inclusive, non-stigmatizing, and effective. The most important elements include the absence of a power difference between the program facilitators (e.g., psychiatrists, fitness coaches) and the participants, an inviting environment to share experiences without feeling judged, peer-to-peer support, a location that is close to home, a variety of activity options for different physical levels, and the opportunity to forge social connections with the other participants. Those designing a physical activity program for individuals with a mood disorder should take into consideration these suggestions and Vancampfort et al.’s [39] ten questions.

Lastly, our findings show that individuals with a mood disorder appreciate the opportunity of staying engaged with the community as a strategy to help with their mental as well as physical health. It is also very important to have access to a stigma-free and non-judgmental environment in order to facilitate fear-free participation. Many participants voiced their concerns of lack of financial resources to enroll in an exercise program, therefore a free or low-cost program will be essential. Overall, we conclude that there is a strong need for a community physical activity program for individuals with a mood disorder and they are best situated to advise on the design and implementation of this program.

### Limitations

Due to their self-selection for the research study, participants might not be representative of the larger population who meet the inclusion/exclusion criteria. The participants who volunteered in our study were of an older age which may have influenced their responses on different aspects of an ideal physical activity program including their preferred intensity (low to moderate). Participants were aware that the project was focused on physical activity and this might have influenced their decision to participate and/or their answers. Participants might already have an appreciation for and/or previous involvement with physical activity, which could have led to biased responses. To reduce these biases, the research team engaged in repeated discussions of the results, used peer researchers for the interviews, and conducted member checking to solicit feedback on the findings from the participants. We believe that the results represent the true needs and perspectives of the participants.

## Conclusion

This study underscores specific factors that can support individuals with a mood disorder to further their health and to increase engagement with physical activity. These factors are relevant to community mental health care and the proven benefits of utilizing a social network approach to solve problems related to a mood disorder. Our evidence suggests that we need to incorporate the perspectives and preferences of the potential participants into the design of a physical activity promotion program. Our findings also point to the benefits of incorporating nature into such a program. The inclusion of opportunities to connect with nature is also consistent with emerging research on the benefits of access to blue and green spaces. Our next step is to design and implement a physical activity program with our peer researchers and then to evaluate the outcomes and effectiveness of the program.
